# PrP^C^-Neutralizing Antibody Confers an Additive Benefit in Combination with 5-Fluorouracil in KRAS-Mutant Colorectal Cancer Models, Associated with Reduced RAS-GTP and AKT/ERK Phosphorylation

**DOI:** 10.3390/ijms27031159

**Published:** 2026-01-23

**Authors:** Jeong Kun Lee, Jun Young Yoon, Jae Young Lee, Sang Hun Lee

**Affiliations:** Program in Biomedical Science & Engineering, Department of Biomedical Sciences, College of Medicine, Inha University, 3-ga, Sinheung-dong, Jung-gu, Incheon 22332, Republic of Korea; jeongkun@inha.ac.kr (J.L.); yoon981008@inha.edu (Y.J.); brookin@inha.edu (L.J.Y.)

**Keywords:** cellular prion protein, colorectal cancer, KRAS mutation, 5-fluorouracil, laminin receptor (RPSA), AKT signaling, tumor angiogenesis

## Abstract

Colorectal cancer (CRC) remains a major cause of cancer-related deaths in advanced disease, and activating KRAS/NRAS mutations limit the use of anti-EGFR antibodies to RAS–wild-type tumors. The cellular prion protein (PrP^C^) has been linked to aggressive and chemoresistant CRC, but its extracellular partners and functional relevance in KRAS-mutant disease are not fully defined. Here, we examined extracellular PrP^C^ complexes and PrP^C^-associated signaling in CRC cell lines and xenografts using a neutralizing PrP^C^ monoclonal antibody. Across a CRC panel that included SNU-C5/WT and its 5-fluorouracil- and oxaliplatin-resistant derivatives, HT-29 (KRAS–wild-type), and HCT-8 and LoVo (KRAS-mutant), co-immunoprecipitation showed that PrP^C^ forms complexes with the 37/67 kDa laminin receptor (RPSA), with PrP^C^–RPSA association particularly increased in KRAS-mutant HCT-8 and LoVo cells. PrP^C^ protein levels were higher in KRAS-mutant HCT-8, SW620, and SNU-407 cells than in HT-29, and PrP^C^ neutralization reduced viability in all four lines. Accordingly, we assessed upstream RAS activity and found that active RAS (RAS-GTP) was higher in KRAS-mutant cells than in HT-29, and PrP^C^ treatment was associated with reduced RAS-GTP levels. In the same KRAS-mutant setting, basal AKT phosphorylation exceeded that in HT-29, and PrP^C^ treatment lowered AKT phosphorylation without changing total AKT. Moreover, PrP^C^ treatment was associated with reduced ERK1/2 phosphorylation in KRAS-mutant cells, suggesting attenuation of downstream RAS pathway output. These signaling changes coincided with a decrease in the S-phase fraction and an increase in G1. In an HCT-8 (KRAS G13D) xenograft model, PrP^C^ monotherapy inhibited tumor growth in a dose-dependent manner, and 5-fluorouracil (5-FU) monotherapy produced an intermediate effect. The combination of PrP^C^ (10 mg/kg) and 5-FU (20 mg/kg) yielded the greatest tumor growth inhibition among the tested regimens. Consistent with this enhanced tumor control, immunofluorescence of xenograft tissues showed that PrP^C^, particularly with 5-FU, reduced intratumoral PrP^C^ and PCNA and decreased CD31-positive microvessels and α-SMA–positive vessel structures. Taken together, these findings suggest that extracellular PrP^C^ supports RAS–AKT signaling, proliferation, and tumor-associated angiogenesis in KRAS-mutant colorectal cancer, and that PrP^C^ neutralization additively enhances 5-fluorouracil activity in KRAS-mutant models. The data provide a preclinical basis for evaluating PrP^C^ antibodies in combination with fluoropyrimidine-based regimens in patients with KRAS-mutant CRC.

## 1. Introduction

Colorectal cancer (CRC) remains a leading cause of cancer-related deaths in patients with unresectable or metastatic disease. Contemporary first-line therapy relies on fluoropyrimidine-based doubles such as folinic acid, fluorouracil, and oxaliplatin (FOLFOX) or folinic acid, fluorouracil, and irinotecan (FOLFIRI), combined with biological agents selected according to molecular features and primary tumor sidedness. Despite these standards, primary and acquired resistance remain prevalent, and activating Kirsten or neuroblastoma rat sarcoma viral oncogene homolog (KRAS/NRAS) mutations render anti-EGFR antibodies ineffective, limiting their use to RAS–wild-type disease. These constraints underscore the need for strategies that can strengthen fluoropyrimidine activity in KRAS-mutant CRC as well as in RAS–wild-type tumors [1,2,3].

The cellular prion protein (PrP^C^) is a glycosylphosphatidylinositol (GPI)-anchored surface glycoprotein associated with several cancer hallmarks, including proliferation, invasion, therapy resistance, and stem-like behavior. In CRC, PrP^C^ expression is enriched in adverse biological states and has been linked to chemoresistant phenotypes. Our previous work, together with studies from other groups, has shown that hypoxic stress upregulates PrP^C^ and augments stemness programs, indicating that PrP^C^ can support tumor persistence during prolonged or repeated exposure to anticancer treatment [4,5,6].

At the plasma membrane, extracellular PrP^C^ has been shown to co-immunoprecipitate and/or colocalize with the 37/67 kDa laminin receptor (RPSA) in CRC models and other systems [4,5,7,8]. These observations support a membrane-proximal organizing role in which PrP^C^ helps coordinate receptor-associated signaling and facilitates downstream activation of pathways such as MAPK/ERK and PI3K/AKT [4,5,6,8,9]. Because these receptor systems converge on RAS-proximal signaling, neutralizing extracellular PrP^C^ has the potential to change pathway throughput even in the presence of KRAS mutations, and thereby to improve the efficacy of fluoropyrimidines in settings where anti-EGFR therapy is ineffective.

On this basis, we hypothesized that blocking extracellular PrP^C^ would additively enhance the antitumor activity of 5-fluorouracil (5-FU) in KRAS-mutant colorectal cancer by attenuating RAS–AKT-ERK signaling and limiting proliferation and angiogenesis. To address this, we (i) mapped PrP^C^ expression and its association with the laminin receptor RPSA across a CRC cell-line panel selected to reflect clinically relevant contexts, including KRAS–wild-type and KRAS-mutant lines, (ii) examined the effect of a neutralizing PrP^C^ C monoclonal antibody on short-term viability, AKT phosphorylation, and cell-cycle distribution in these models, and (iii) evaluated PrP^C^, alone and in combination with 5-FU, in an HCT-8 (KRAS G13D) xenograft model, including analyses of intratumoral proliferation (PCNA) and tumor-associated vasculature (CD31 and α-SMA). These experiments were designed to clarify how extracellular PrP^C^ contributes to RAS–AKT pathway activity and to provide preclinical evidence that PrP^C^ neutralization has therapeutic potential in KRAS-mutant CRC treated with fluoropyrimidine-based regimens.

## 2. Results

### 2.1. Co-Immunoprecipitation of PrP^C^–RPSA Complexes Across Colorectal Cancer Cell Lines

**Figure 1:**
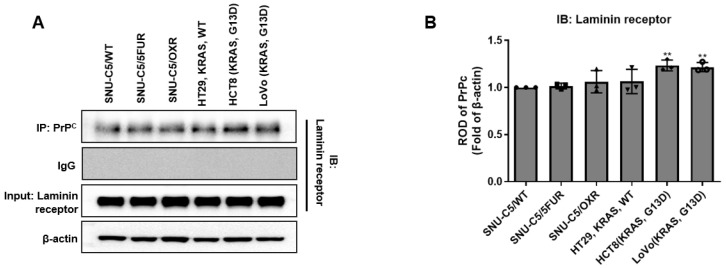
Co-immunoprecipitation of PrP^C^ with the laminin receptor RPSA in colorectal cancer cell lines. (**A**) Cell lysates from colorectal cancer (CRC) cell lines (SNU-C5/WT, SNU-C5/5FUR, SNU-C5/OXR, HT-29, HCT-8, and LoVo) were subjected to immunoprecipitation (IP) with a neutralizing PrP^C^ antibody or isotype-matched IgG, followed by immunoblotting (IB) for the laminin receptor RPSA (37/67 kDa). PrP^C^ in the immunoprecipitates and β-actin in the input lysates are shown as IP and loading controls, respectively. Representative of at least three independent experiments. (**B**) Densitometric quantification of RPSA co-immunoprecipitation signals normalized to PrP^C^ in the corresponding immunoprecipitates. Values are expressed relative to one reference line and shown as mean ± SEM from ≥3 independent experiments. CRC, colorectal cancer; PrP^C^, cellular prion protein; RPSA, 37/67 kDa laminin receptor. Statistics: one-way ANOVA with Tukey’s test; ** *p* < 0.01 vs. SNU-C5WT.

To examine whether PrP^C^ forms complexes with the 37/67 kDa laminin receptor RPSA in our predefined colorectal cancer (CRC) panel, we performed co-immunoprecipitation using a neutralizing PrP^C^ antibody and matched isotype IgG controls (Figure 1A). RPSA was detected in PrP^C^ immunoprecipitates from all six CRC lines, with the strongest bands in the KRAS-mutant lines HCT-8 and LoVo and weaker signals in the SNU-C5 derivatives and HT-29. Densitometric analysis of RPSA signals normalized to PrP^C^ in the corresponding immunoprecipitates (Figure 1B) confirmed that PrP^C^–RPSA binding was enriched in HCT-8 and LoVo compared with the SNU-C5 sublines and HT-29. Because HCT-8 and LoVo harbor activating KRAS mutations, this preferential PrP^C^–RPSA association in KRAS-mutant cells suggests a potential relationship between the PrP^C^–RPSA scaffold and KRAS-mutant signaling states; however, co-immunoprecipitation alone does not establish functional mediation of downstream pathways.

These data indicate that PrP^C^–RPSA complexes are a shared feature of the CRC lines used in this study and provide a molecular context for subsequent experiments with the PrP^C^ antibody. Accordingly, we next assessed RAS activity (RAS-GTP) and downstream effector signaling, including AKT and ERK phosphorylation, following PrP^C^ neutralization.

### 2.2. Neutralizing PrP^C^ Preferentially Reduces Viability in KRAS-Mutant Colorectal Cancer Cells

**Figure 2:**
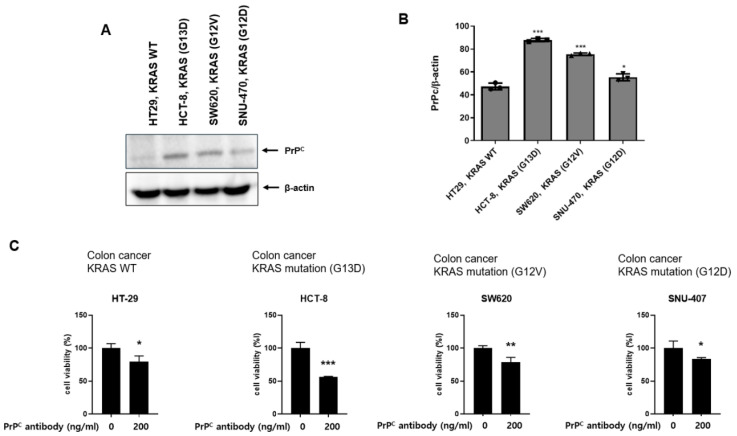
PrP^C^ expression and viability response to PrP^C^ neutralization in colorectal cancer cell lines. (**A**) Western blot analysis of PrP^C^ in KRAS–wild-type HT-29 and KRAS-mutant HCT-8, SW620, and SNU-407 colorectal cancer cell lines. β-actin serves as a loading control. (**B**) Densitometric quantification of PrP^C^ protein levels from the Western blot. Values are expressed relative to one reference line and shown as mean ± SEM from ≥3 independent experiments. Statistics: one-way ANOVA with Tukey’s test. (**C**) Effects of the neutralizing PrP^C^ antibody (200 ng/mL, 24 h) on cell viability measured by MTT assay in HT-29, HCT-8, SW620, and SNU-407. Viability is expressed relative to the untreated control for each cell line and shown as mean ± SEM from at least three independent experiments; * *p* < 0.05, ** *p* < 0.01 and *** *p* < 0.001 vs. control.

To examine whether PrP^C^ expression is linked to KRAS mutation status, we compared PrP^C^ levels between KRAS–wild-type HT-29 and KRAS-mutant HCT-8, SW620, and SNU-407 colorectal cancer cell lines by Western blotting. PrP^C^ protein was detectable in all lines, but its expression was consistently higher in the KRAS-mutant HCT-8, SW620, and SNU-407 cells than in HT-29 (Figure 2A). Treatment with the neutralizing PrP^C^ monoclonal antibody (clone CSA0156, 200 ng/mL) for 24 h significantly reduced MTT-based viability in HT-29, HCT-8, SW620, and SNU-407, with a larger reduction in the KRAS-mutant HCT-8, SW620, and SNU-407 cells than in KRAS–wild-type HT-29 (Figure 2B). These findings suggest that cells with higher PrP^C^ expression, such as the KRAS-mutant HCT-8, SW620, and SNU-407 lines, are more reliant on PrP^C^ for survival and are consistent with the possibility that increased PrP^C^ is accompanied by more active downstream RAS signaling in the KRAS-mutant setting.

### 2.3. PrP^C^ Treatment Is Associated with Reduced RAS Activity (RAS-GTP) in KRAS-Mutant Colorectal Cancer Cells

**Figure 3:**
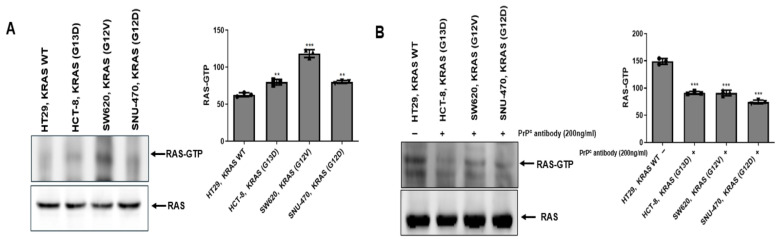
RAS activation (RAS-GTP) in KRAS–wild-type and KRAS-mutant colorectal cancer cells and the effect of PrP^C^ neutralization. (**A**) RAS-GTP pull-down assay comparing active RAS (RAS-GTP) levels in KRAS–wild-type HT-29 and KRAS-mutant HCT-8, SW620, and SNU-407 colorectal cancer cell lines under baseline conditions. Representative immunoblots show RAS-GTP and total RAS. (**B**) RAS-GTP pull-down assay in HT-29, HCT-8, SW620, and SNU-407 cells after treatment with PrP^C^ antibody (200 ng/mL, 24 h) or vehicle. Representative immunoblots show RAS-GTP and total RAS. Densitometry values were normalized to the HT29 cell line within each cell line and are presented as mean ± SEM from at least three independent experiments; ** *p* < 0.01 and *** *p* < 0.001 vs. HT29.

To assess whether PrP^C^ blockade is linked to upstream RAS activation, we measured active RAS (RAS-GTP) using a RAS activation pull-down assay in KRAS–wild-type HT-29 and KRAS-mutant HCT-8, SW620, and SNU-407 cells (Figure 3A). Under basal conditions, RAS-GTP signals were higher in the KRAS-mutant cell lines than in HT-29, consistent with a more activated RAS state in the mutant background. We then re-assessed RAS activity following treatment with the neutralizing PrP^C^ antibody and observed a reduction in RAS-GTP signals compared with vehicle-treated controls (Figure 3B), indicating that PrP^C^ treatment is associated with decreased RAS activation status.

### 2.4. PrP^C^ Antibody Attenuates AKT Activation in KRAS-Mutant Colorectal Cancer Cells

**Figure 4:**
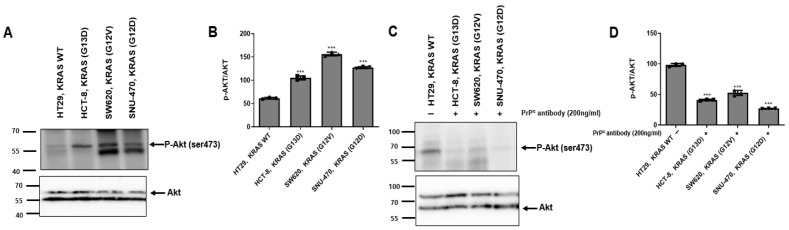
AKT activation in KRAS–wild-type and KRAS-mutant colorectal cancer cells and its modulation by PrP^C^ neutralization. (**A**) Western blot analysis of phosphorylated AKT (p-AKT) and total AKT in KRAS–wild-type HT-29 and KRAS-mutant HCT-8, SW620, and SNU-407 colorectal cancer cell lines under basal conditions. β-actin serves as a loading control. (**B**) Densitometric quantification of p-AKT/AKT ratios from the Western blot shown in (**A**). Values were normalized to HT-29 and are presented as mean ± SEM from at least three independent experiments. Statistics: one-way ANOVA with Tukey’s test; *** *p* < 0.001 vs. HT29. (**C**) Western blot analysis of p-AKT and total AKT in HT-29, HCT-8, SW620, and SNU-407 after treatment with the neutralizing PrP^C^ antibody (200 ng/mL, 24 h) or vehicle control. β-actin is shown as a loading control. (**D**) Values were normalized to HT-29 and are presented as mean ± SEM from at least three independent experiments. Statistics: one-way ANOVA with Tukey’s test; *** *p* < 0.001 vs. HT29.

To assess whether PrP^C^ neutralization is associated with changes in AKT activation, we examined AKT phosphorylation and total AKT by Western blotting (Figure 4A). In untreated cells, the ratio of phosphorylated AKT to total AKT was higher in the KRAS-mutant HCT-8, SW620, and SNU-407 cells than in HT-29 cells, indicating elevated basal AKT pathway activity in the mutant lines. When the neutralizing PrP^C^ antibody was applied, AKT phosphorylation was reduced in the KRAS-mutant cells, whereas total AKT levels were largely unchanged (Figure 4C). Taken together, these findings suggest that PrP^C^ contributes to the maintenance of RAS–AKT signaling in KRAS-mutant colorectal cancer cells and may participate in driving proliferative signaling in this setting.

### 2.5. PrP^C^ Antibody Attenuates ERK Activation in KRAS-Mutant Colorectal Cancer Cells

**Figure 5:**
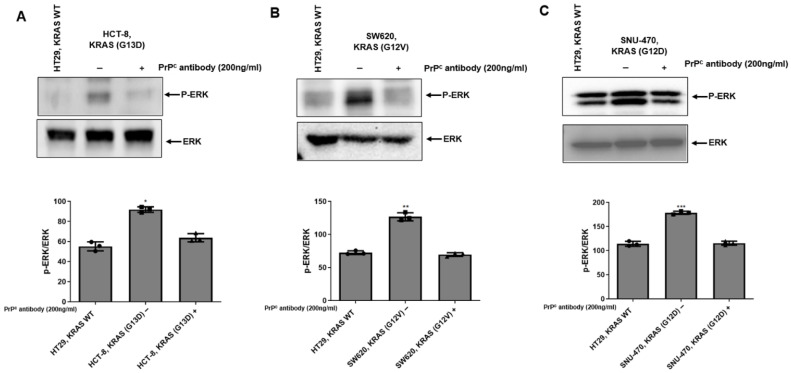
PrP^C^ antibody decreases ERK1/2 phosphorylation in KRAS-mutant colorectal cancer cells. (**A**–**C**) Immunoblot analysis of phosphorylated ERK1/2 (p-ERK) and total ERK in HT-29 (KRAS WT) and KRAS-mutant colorectal cancer cell lines: (**A**) HCT-8 (KRAS G13D), (**B**) SW620 (KRAS G12V), and (**C**) SNU-470 (KRAS G12D). Cells were treated with vehicle (−) or PrP^C^ antibody (200 ng/mL; 24 h). Representative blots are shown. Bar graphs depict densitometric quantification of p-ERK normalized to total ERK (p-ERK/ERK), expressed relative to the vehicle condition within each KRAS-mutant cell line. Statistics: one-way ANOVA with Tukey’s test; * *p* < 0.05, ** *p* < 0.01 and *** *p* < 0.001 vs. HT29.

To further evaluate whether PrP^C^ blockade modulates RAS pathway output beyond AKT, we examined ERK1/2 phosphorylation in KRAS–wild-type HT-29 and KRAS-mutant colorectal cancer cell lines. Under basal conditions, KRAS-mutant HCT-8, SW620, and SNU-470 cells showed higher p-ERK signals compared with HT-29 (Figure 5A–C). Treatment with the PrP^C^ antibody (200 ng/mL, 24 h) reduced p-ERK levels across the KRAS-mutant cell lines, while total ERK levels were largely maintained (Figure 5A–C). These results support that PrP^C^ neutralization in KRAS-mutant colorectal cancer cells is associated with reduced phosphorylation of AKT and ERK downstream of the RAS pathway.

### 2.6. PrP^C^ Neutralization Decreases S-Phase Entry in KRAS-Mutant Colorectal Cancer Cells

**Figure 6:**
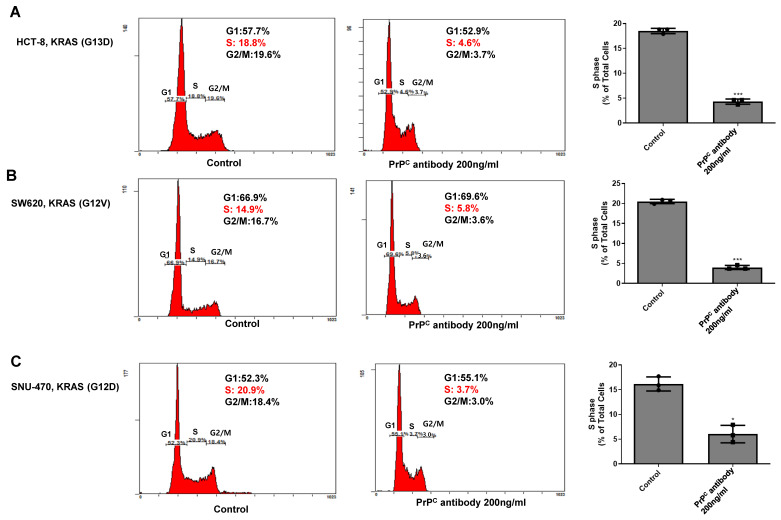
PrP^C^ neutralization reduces S-phase fraction in KRAS-mutant colorectal cancer cells. (**A**–**C**) Representative PI-based DNA content histograms of KRAS-mutant HCT-8, SW620, and SNU-407 colorectal cancer cells treated with vehicle (Control) or the neutralizing PrP^C^ antibody (200 ng/mL, 24 h). The percentages of cells in G1, S, and G2/M phases are indicated in each plot. Quantification of S-phase fractions in HCT-8, SW620, and SNU-407 cells corresponding to the experiments shown in (**A**–**C**). Bars represent mean ± SEM from at least three independent experiments. Statistics: one-way ANOVA with Tukey’s test; * *p* < 0.05 and *** *p* < 0.001 vs. control.

To assess whether PrP^C^ neutralization affects cell-cycle progression in KRAS-mutant colorectal cancer cells, we performed PI-based flow-cytometric cell-cycle analysis in HCT-8, SW620, and SNU-407 cells with or without the neutralizing PrP^C^ antibody. In untreated cells, the S-phase fraction ranged from approximately 15–20%, consistent with actively cycling populations (Figure 6A–C). After PrP^C^ antibody treatment, the S-phase fraction was markedly reduced to ~4–6% in all three KRAS-mutant lines, with a corresponding increase in the G1 population and only minor changes in G2/M. Quantitative analysis confirmed a significant decrease in S-phase entry across the panel. These data indicate that PrP^C^ neutralization slows proliferation of KRAS-mutant colorectal cancer cells by limiting progression into S phase, and, together with the reduction in RAS-AKT-ERK activation, support a model in which PrP^C^ promotes RAS–AKT pathway activity and thereby sustains cell-cycle progression in these cells.

### 2.7. PrP^C^ Retains Antitumor Activity in a KRAS-Mutant Xenograft and Improves 5-FU Response

**Figure 7:**
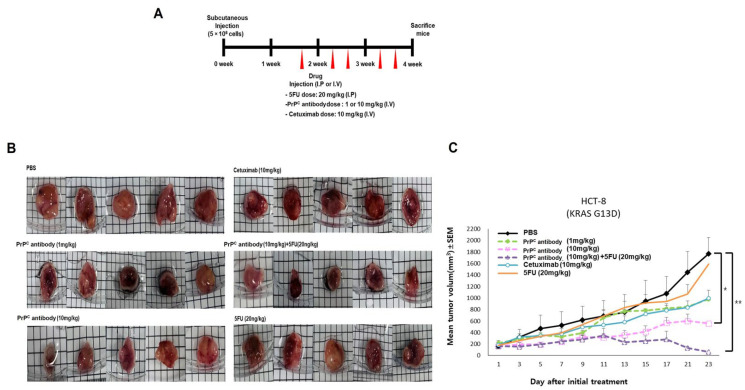
Effect of PrP^C^ antibody and 5-FU in an HCT-8 (KRAS G13D) xenograft model. (**A**) Treatment scheme for subcutaneous HCT-8 (KRAS G13D) xenografts. After tumor establishment (~100 mm^3^), mice were randomized (*n* = 5 per group) to receive PBS, PrP^C^ antibody (PrP^C^ antibody, 1 or 10 mg/kg, i.v.), 5-fluorouracil (5-FU, 20 mg/kg, i.p.), cetuximab (10 mg/kg, i.v.), or PrP^C^ antibody (10 mg/kg, i.v.) plus 5-FU (20 mg/kg, i.p.). Arrows indicate dosing days (twice weekly). (**B**) Representative tumors at the end of treatment from each group shown in (**A**). (**C**) Tumor growth curves for HCT-8 xenografts under the indicated treatments. Tumor length and width were measured three times per week using calipers by assessors blinded to group allocation, and tumor volume was calculated as V = (length × width^2^)/2. Data are presented as mean ± SD (*n* = 5 per group). For statistical analysis, the area under the tumor growth curve (AUC) was calculated for each mouse (trapezoidal method) and compared across groups using one-way ANOVA with Tukey’s multiple-comparisons test; * *p* < 0.05 and ** *p* < 0.01 vs. PBS.

To examine the in vivo effect of PrP^C^ neutralization in a KRAS-mutant background, we used a subcutaneous HCT-8 (KRAS G13D) xenograft model. When tumors reached approximately 100 mm^3^, mice were randomized into treatment groups (*n* = 5 per group), and tumor measurements and data analysis were performed in a blinded manner (Section 4.9). After randomization, mice received twice-weekly treatment, as shown in Figure 7A: PBS, PrP^C^ antibody 1 or 10 mg/kg (i.v.), 5-FU 20 mg/kg (i.p.), cetuximab 10 mg/kg (i.v.), or PrP^C^ antibody 10 mg/kg plus 5-FU 20 mg/kg. Representative tumors from each group are shown in Figure 7B. In this KRAS-mutant model, cetuximab produced only a small change in tumor size, whereas PrP^C^ antibody alone reduced tumor growth in a dose-responsive manner, with 10 mg/kg giving smaller tumors than PBS or 1 mg/kg. 5-FU monotherapy produced an intermediate effect. The combination of PrP^C^ antibody and 5-FU showed the most pronounced inhibition among the tested regimens, and several animals maintained low tumor volumes over the observation period (Figure 7C). These findings indicate that PrP^C^ neutralization remains effective in a KRAS-mutant colorectal cancer xenograft and is consistent with an additive antitumor effect when combined with 5-FU.

### 2.8. PrP^C^ Antibody Reduces Intratumoral PrP^C^ and Proliferating Cells in KRAS-Mutant Xenografts

**Figure 8:**
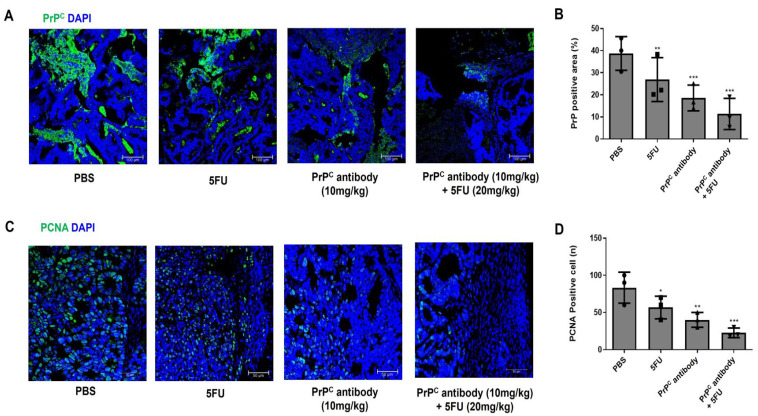
PrP^C^ and PCNA immunofluorescence in HCT-8 (KRAS G13D) xenograft tumors treated with PrP^C^ antibody and 5-FU. (**A**) Representative immunofluorescence images of PrP^C^ in HCT-8 xenograft sections from mice treated with PBS, PrP^C^ antibody (10 mg/kg), 5-FU (20 mg/kg), or PrP^C^ antibody (10 mg/kg) plus 5-FU (20 mg/kg). PrP^C^ (green); nuclei, DAPI (blue). (**B**) Quantification of PrP^C^ immunofluorescence intensity in tumors from the four treatment groups shown in (**A**). Values are expressed relative to the PBS group. Statistics: one-way ANOVA with Tukey’s test; ** *p* < 0.01 and *** *p* < 0.001 vs. PBS. (**C**) Representative immunofluorescence images of PCNA in the same set of tumors. PCNA (green); nuclei, DAPI (blue). (**D**) Quantification of PCNA-positive nuclei in tumors from the four treatment groups shown in (**C**). Statistics: one-way ANOVA with Tukey’s test; * *p* < 0.05, ** *p* < 0.01 and *** *p* < 0.001 vs. PBS.

To examine how PrP^C^-targeted treatment alters intratumoral PrP^C^ and proliferation, we performed immunofluorescence staining for PrP^C^ and PCNA in HCT-8 (KRAS G13D) xenograft tumors from mice treated with PBS, PrP^C^ antibody (10 mg/kg), 5-FU (20 mg/kg), or PrP^C^ antibody (10 mg/kg) plus 5-FU (20 mg/kg). In PBS-treated tumors, PrP^C^ staining was strong along tumor cell nests, whereas PrP^C^ antibody treatment clearly reduced PrP^C^ signal; 5-FU alone produced an intermediate reduction, and the combination group showed the weakest PrP^C^ staining (Figure 8A,B). PCNA staining showed a similar pattern: PBS tumors contained many PCNA-positive nuclei, PrP^C^ antibody or 5-FU alone lowered the number of PCNA-positive cells, and the combination produced the lowest PCNA Labeling, as confirmed by quantitative analysis (Figure 8C,D). PrP^C^ signal and proliferation were quantified as PrP^C^ fluorescence intensity and the percentage of PCNA-positive nuclei among total DAPI-positive nuclei, respectively. These findings show that PrP^C^ neutralization decreases intratumoral PrP^C^ and the proportion of proliferating cells in KRAS-mutant colorectal tumors, and are consistent with additive tumor control in the antibody plus 5-FU group in KRAS-mutant colorectal cancer.

### 2.9. PrP^C^ Neutralization Reduces Tumor-Associated Vessel Formation in KRAS-Mutant Xenografts

**Figure 9:**
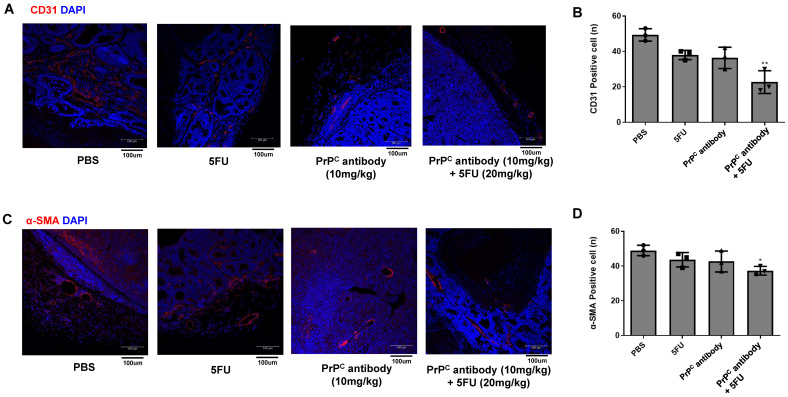
CD31 and α-SMA immunofluorescence in HCT-8 (KRAS G13D) xenograft tumors after treatment with PrP^C^ antibody and 5-FU. (**A**) Representative immunofluorescence images of CD31 in HCT-8 xenograft sections from mice treated with PBS, PrP^C^ antibody (10 mg/kg), 5-FU (20 mg/kg), or PrP^C^ antibody (10 mg/kg) plus 5-FU (20 mg/kg). CD31 (red); nuclei, DAPI (blue). (**B**) Quantification of CD31-positive signals in tumors from the four treatment groups shown in (**A**), expressed relative to the PBS group. Statistics: one-way ANOVA with Tukey’s test; ** *p* < 0.01 vs. PBS. (**C**) Representative immunofluorescence images of α-SMA in the same set of tumors. α-SMA (red); nuclei, DAPI (blue). (**D**) Quantification of α-SMA–positive vessel structures in tumors from the four treatment groups shown in (**C**). Statistics: one-way ANOVA with Tukey’s test; * *p* < 0.05 vs. PBS.

To assess whether PrP^C^-targeted treatment affects tumor vasculature, we performed immunofluorescence staining for the endothelial marker CD31 and the vascular smooth muscle marker α-SMA in HCT-8 (KRAS G13D) xenograft tumors from mice treated with PBS, PrP^C^ antibody (10 mg/kg), 5-FU (20 mg/kg), or PrP^C^ antibody (10 mg/kg) plus 5-FU (20 mg/kg). CD31 staining showed dense networks of microvessels in PBS tumors, whereas PrP^C^ antibody or 5-FU alone reduced the extent of CD31-positive structures; the combination group displayed the lowest CD31 signal among the four regimens (Figure 9A,B). α-SMA staining, reflecting more mature arteriolar-type vessels, also decreased in the PrP antibody and 5-FU groups and was least prominent in tumors from the combination group (Figure 9C,D). These findings indicate that PrP^C^ neutralization, particularly when combined with 5-FU, is associated with reduced microvessel and arterial-like vessel formation in KRAS-mutant colorectal tumors, suggesting that inhibition of tumor-associated angiogenesis is one way in which PrP^C^ blockade can limit tumor growth.

## 3. Discussion

PrP^C^ is implicated in proliferation, therapeutic resistance, metastatic spread, and stem-like traits across multiple cancers, including CRC [10,11,12]. In CRC, several reports indicate that extracellular PrP^C^ can interact with the 37/67 kDa laminin receptor (RPSA) at the cell surface [4,5,6,8,9], suggesting that PrP^C^ may act as a regulator of receptor signaling at the plasma membrane. On this basis, it is reasonable to consider that PrP^C^ may influence the response to fluoropyrimidine-based therapy across different KRAS backgrounds. In the present study, co-immunoprecipitation across a predefined CRC panel confirmed that PrP^C^ forms complexes with RPSA and showed that PrP^C^–RPSA association is particularly enriched in KRAS-mutant HCT-8 and LoVo cells. However, the present study does not test whether RPSA is required for the PrP^C^-associated signaling changes; therefore, RPSA is discussed here as a candidate mediator rather than a confirmed functional effector. We further observed that PrP^C^ protein levels were higher in KRAS-mutant HCT-8, SW620, and SNU-407 cells than in KRAS–wild-type HT-29 cells, and that PrP^C^ neutralization reduced short-term viability in all four lines, with a larger decrease in the KRAS-mutant cells. These findings indicate that PrP^C^ contributes to cell-surface signaling in CRC and that KRAS-mutant cells show a greater functional reliance on PrP^C^ for survival.

PrP^C^ has been proposed as a GPI-anchored scaffold that can associate with receptor assemblies (including EGFR/c-MET and RPSA) and may modulate downstream MAPK/ERK and PI3K/AKT signaling, epithelial–mesenchymal transition, and stem-like programs [4,5,6,10,11,13,14]. In cancer biology, “prion-like” behavior does not refer to infectious templating but rather to stress-induced upregulation and clustering in membrane microdomains that sustain signaling complexes [15]. Under hypoxia and other tumor-related stresses, PrP^C^ abundance is further shaped by protein-quality control pathways such as HIF-1α/HSPA1L-dependent stabilization and GP78-linked ubiquitination/proteasomal turnover, which have been implicated in the regulation of PrP^C^ levels and hypoxia/TRAIL tolerance [12,16,17,18]. Within this framework, our data indicate that PrP^C^ expression and PrP^C^–RPSA binding are increased in KRAS-mutant cells. In addition, upstream RAS activity was higher in KRAS-mutant cells than in KRAS–wild-type HT-29, and PrP^C^ antibody treatment was associated with reduced RAS-GTP levels. Consistent with this, basal AKT phosphorylation was elevated in KRAS-mutant cells, and PrP^C^ antibody treatment lowered AKT phosphorylation without materially altering total AKT. Moreover, PrP^C^ antibody treatment was associated with reduced ERK1/2 phosphorylation (p-ERK/ERK) in KRAS-mutant cells, suggesting attenuation of downstream RAS pathway signaling. These findings support a model in which PrP^C^–RPSA complexes help maintain upstream receptor/adhesion inputs that promote Ras activation, and PrP^C^ neutralization reduces the total Ras-GTP pool, accompanied by decreased AKT and ERK1/2 phosphorylation.

Similar patterns were observed in vivo in a KRAS-mutant xenograft model. In HCT-8 (KRAS G13D) xenografts, PrP^C^ antibody monotherapy reduced tumor growth in a dose-dependent manner, whereas 5-FU monotherapy produced an intermediate level of control. The combination of PrP^C^ and 5-FU produced the most pronounced growth inhibition among the regimens tested, whereas cetuximab plus 5-FU had only modest effects in this KRAS-mutant model. Immunofluorescence analyses of xenograft tissues provided additional support at the tissue level: PrP^C^, particularly in combination with 5-FU, lowered intratumoral PrP^C^ and PCNA and reduced CD31-positive microvessels and α-SMA–positive arteriolar structures. These findings suggest that PrP^C^ blockade limits tumor-cell proliferation and tumor-associated angiogenesis, consistent with the additive tumor control observed with the antibody plus 5-FU.

These data have direct implications for therapeutic strategy. The clinical benefit of anti-EGFR therapy is restricted by RAS status; activating KRAS/NRAS mutations abrogate efficacy, limiting EGFR antibodies to RAS–wild-type disease [1,2,3]. By contrast, in our models the PrP^C^ antibody reduced viability in both KRAS–wild-type and KRAS-mutant CRC cells and retained antitumor activity in a KRAS-mutant xenograft, where its combination with 5-FU led to greater tumor control than that observed with cetuximab plus 5-FU. Targeting PrP^C^-associated receptor complexes at the cell surface may therefore bypass some KRAS-linked resistance mechanisms while remaining compatible with fluoropyrimidine-based regimens such as FOLFOX and FOLFIRI [2,3]. Early-phase clinical development of PrP^C^ should include RAS-mutant cohorts and incorporate receptor-proximal readouts, such as phosphorylation of EGFR/c-MET and downstream AKT/ERK, to clarify how PrP^C^ blockade modulates signaling in patient tumors.

This study has limitations. All experiments were conducted in established cell lines and subcutaneous xenografts; orthotopic and patient-derived models will be important to better capture the heterogeneity of human CRC. The cell-cycle and immunofluorescence analyses were carried out in selected KRAS-mutant lines and a single xenograft model, and extension to additional genetic backgrounds would strengthen the generalizability of our conclusions. We did not define the precise epitope and binding kinetics of the antibody in this work, nor did we characterize Fc-effector functions or perform GLP toxicology. Future studies should address these points, include more detailed mapping of receptor-proximal phosphorylation after PrP^C^ blockade, and evaluate combination therapy with current standard regimens in models that incorporate chemotherapy plus targeted agents.

## 4. Materials and Methods

### 4.1. Reagents and Antibodies

Reagents included 5-fluorouracil (5-FU; Sigma-Aldrich, St. Louis, MO, USA) and cetuximab (Sigma-Aldrich, St. Louis, MO, USA). and cetuximab (commercially obtained according to the manufacturer’s instructions). The neutralizing PrP^C^ monoclonal antibody (human IgG1, κ), hereafter referred to as PrP^C^ antibody, was generated under a fee-for-service contract by Y-Biologics Co., Ltd. (Daejeon, Republic of Korea), expressed in mammalian cells, and purified by Protein A affinity chromatography. The antibody was formulated in phosphate-buffered saline (PBS, pH 7.4) and met research-grade specifications (endotoxin ≤ 1 EU/mg; monomer > 95% by size-exclusion chromatography). An isotype-matched human IgG1-κ (Y-Biologics) was used as a control. Working concentrations for in vitro assays were determined from preliminary titration experiments. PrP^C^ antibody was used at 200 ng/mL for viability and cell-cycle analyses. For in vivo studies with HCT-8 (KRAS G13D) xenografts, PrP antibody was administered at 1 or 10 mg/kg by intravenous injection twice weekly, and 5-FU at 20 mg/kg by intraperitoneal injection, according to the treatment schedules described in the xenograft section.

### 4.2. Epitope Mapping

Epitope mapping was performed through high-density peptide array scanning of human PrP^C^ PrP antibody targets a linear epitope centered on WNKPSK (residues 99–104) (Supplementary Figure S1).

### 4.3. Cell Culture

The SNU-C5 cell lines—wild-type (SNU-C5/WT), oxaliplatin-resistant (SNU-C5/OXR), and 5-fluorouracil-resistant (SNU-C5/5FUR)—were obtained from the Research Center for Resistant Cells at Chosun University (Gwangju, Republic of Korea). The colorectal cancer cell lines HT-29, HCT-8, LoVo, SW620, and SNU-407 were purchased from the Korean Cell Line Bank (KCLB; Seoul, Republic of Korea).

HT-29, LoVo, and HCT-8 cells were maintained in Dulbecco’s modified Eagle’s medium (DMEM; 4.5 g/L glucose) supplemented with 10% fetal bovine serum (FBS), L-glutamine, and penicillin/streptomycin at 37 °C in a humidified incubator with 5% CO_2_. SNU-C5 derivatives (WT, 5FUR, and OXR), SW620, and SNU-407 were maintained in RPMI-1640 medium with 10% FBS under the same conditions. SNU-C5/5FUR cells were routinely pulsed with 140 μM 5-FU for 2 days, followed by incubation in drug-free medium for 4 days before use in experiments. All cell lines were authenticated, routinely tested for mycoplasma contamination, and used at low passage numbers.

### 4.4. Co-Immunoprecipitation and Immunoblotting

Cells (~1–2 × 10^7^) were lysed on ice for 30 min in NP-40 buffer (50 mM Tris-HCl, pH 7.5, 150 mM NaCl, 1% NP-40, and protease/phosphatase inhibitors) and clarified at 14,000× *g* for 10 min. After pre-clearing, 1–2 mg of total protein was incubated overnight at 4 °C with PrP^C^ or isotype control IgG and captured with Protein G agarose (2 h). Beads were washed five times with lysis buffer and eluted in Laemmli sample buffer. Inputs (5–10%) and immunoprecipitates were resolved via SDS-PAGE and immunoblotted for RPSA; β-actin in the input lysates served as a loading control. Co-immunoprecipitation was repeated across the CRC panel (SNU-C5/WT, 5FUR, OXR; HT-29, HCT-8, and LoVo). Where indicated, densitometry was performed in a blinded manner using identical exposure settings across groups.

### 4.5. Cell Viability Assay with PrP^C^ Antibody

For the assessment of viability, cells were seeded in 96-well plates at a density of 3–6 × 10^3^ cells per well and treated with PrP^C^ antibody (200 ng/mL) for 24 h. Viability was quantified using a 3-(4,5-dimethylthiazol-2-yl)-2,5-diphenyltetrazolium bromide (MTT) assay, and formazan absorbance was measured at 570–575 nm. The colorectal cancer (CRC) cell lines HT-29, HCT-8, SW620, and SNU-407 were included in this analysis. The single-dose viability data reflect pre-specified working concentrations derived from preliminary titration experiments and were used for cross-condition comparison rather than full dose–response modeling.

### 4.6. Ras Activity Assay

Levels of active, GTP-bound Ras (Ras-GTP) were determined using a GST–RAF1-RBD pull-down assay (Thermo Scientific, Waltham, MA, USA). Briefly, GST–RAF1-RBD fusion proteins were incubated with glutathione beads to immobilize the RBD. Cell lysates were prepared in the lysis buffer provided by the manufacturer’s protocol and incubated with the GST–RAF1-RBD–immobilized beads for 1.5 h at 4 °C. Bound proteins were eluted with SDS sample buffer and analyzed by Western blot using an anti-Ras antibody; Ras signals in the pull-down fraction were interpreted as Ras-GTP.

### 4.7. Western Blot Analysis

Cells were lysed and total cellular protein was extracted using RIPA lysis buffer (Thermo-Fisher Scientific). Cell lysates were then subjected to sodium dodecyl sulfate-polyacryl-amide gel electrophoresis (SDS-PAGE), and proteins were transferred to polyvinylidenefluoride membranes (PVDFs; Millipore, Billerica, MA, USA). The membranes were blocked with 5% skim milk and incubated with primary antibodies against cellular prion protein (PrP^C^), p-AKT, AKT, pERK, ERK, and β-actin (Santa Cruz Biotechnology, Santa Cruz, CA, USA). After incubation of the membranes with peroxidase-conjugated secondary antibodies (Santa Cruz Biotechnology), bands were visualized using enhanced chemiluminescence reagents (Amersham Biosciences, Uppsala, Sweden) in a dark room.

### 4.8. Cell Cycle Analysis and Dose Selection for Combination Studies

For PI–FACS analysis, cells were treated with PrP^C^ antibody (200 ng/mL) or vehicle control for 24 h, fixed in 70% ethanol (−20 °C, ≥2 h), treated with RNase A (100 µg/mL), and stained with propidium iodide (50 µg/mL). Data were acquired on a BD flow cytometer and analyzed with ModFit to determine the G1/S/G2–M fractions. Gating boundaries and *x*-axis scales were kept identical across all groups, sample identifiers were blinded during analysis, and ModFit parameters were held constant for all runs.

### 4.9. Xenograft Studies (HCT-8 KRAS G13D)

HCT-8 colorectal cancer cells (KRAS G13D; 5 × 10^6^ cells in 100 µL PBS) were subcutaneously implanted in the flanks of male BALB/c nude mice (8–10 weeks old). When tumors reached approximately 100 mm^3^, animals were randomized (stratified by baseline tumor volume) into six treatment groups (*n* = 5 per group) and dosed twice weekly (every 3–4 days) as follows: PBS vehicle; PrP antibody 1 mg/kg i.v.; PrP^C^ antibody 10 mg/kg i.v.; 5-fluorouracil (5-FU) 20 mg/kg i.p.; cetuximab 10 mg/kg i.v.; or PrP^C^ antibody 10 mg/kg i.v. plus 5-FU 20 mg/kg i.p. Tumor length (a) and width (b) were measured three times per week with a Vernier caliper, and tumor volume was calculated as V = (a × b^2^)/2. Body weight was monitored throughout the study to assess general tolerability. Humane endpoints and euthanasia followed institutional animal care guidelines. Outcome assessors were blinded to group allocation during tumor measurement and data analysis, and all randomized animals were included in the analysis unless euthanized early due to humane endpoints.

### 4.10. Immunofluorescence Staining

For histological analysis, tumors from HCT-8 (KRAS G13D) xenografts were fixed in 4% paraformaldehyde (Affymetrix, Santa Clara, CA, USA) and embedded in paraffin. Sections were incubated with primary antibodies against PrP^C^, proliferating cell nuclear antigen (PCNA), CD31, and α-smooth muscle actin (α-SMA) (Santa Cruz Biotechnology). After washing, sections were incubated with appropriate Alexa Fluor 488– or Alexa Fluor 594–conjugated secondary antibodies (Thermo Fisher Scientific). Nuclei were counterstained with 4′,6-diamidino-2-phenylindole (DAPI; Sigma-Aldrich). Immunofluorescence images were acquired using a confocal microscope (Leica, Wetzlar, Germany).

### 4.11. Statistics

Data are presented as mean ± standard error of the mean (SEM). Comparisons between two groups were performed using two-tailed unpaired Student’s *t*-tests. Multiple-group comparisons were analyzed by one-way or two-way analysis of variance (ANOVA) with appropriate post hoc tests (Dunnett or Tukey). For longitudinal tumor-volume curves, time × treatment interactions were assessed using two-way repeated-measures ANOVA (or a mixed-effects model when missing values occurred). A *p* value < 0.05 was considered statistically significant. Sample sizes are provided in the figure legends. Analyses were performed with blinding to group allocation whenever feasible.

### 4.12. Ethics

All animal procedures were conducted at the Preclinical Research Center, Healthcare Innovation Park, Seoul National University Bundang Hospital (SNUBH) in accordance with institutional guidelines and were approved by the Institutional Animal Care and Use Committee (IACUC) of SNUBH (Approval No. BA-2203-339-002-02). No human subjects or identifiable human materials were used.

## 5. Conclusions

These results suggest that extracellular PrP^C^ is associated with RPSA-related signaling and RAS pathway activity in CRC, and that both PrP^C^ expression and PrP^C^–RPSA association are increased in KRAS-mutant cells. Consistent with this, active RAS (RAS-GTP) was higher in KRAS-mutant cells than in KRAS–wild-type cells, and PrP^C^ antibody treatment was associated with reduced RAS-GTP levels. Moreover, PrP^C^ neutralization was associated with reduced AKT phosphorylation and reduced ERK1/2 phosphorylation (p-ERK), accompanied by decreased S-phase entry and reduced proliferation in vitro. In a KRAS-mutant xenograft, PrP^C^ neutralization improved tumor control, and the antibody plus 5-FU regimen showed greater tumor growth inhibition than either monotherapy, while reducing PCNA and vascular markers (CD31 and α-SMA). Collectively, these findings provide a rationale for evaluating PrP^C^-neutralizing antibodies in combination with fluoropyrimidine-based regimens, including in KRAS-mutant colorectal cancer.

## Data Availability

The data that support the findings of this study are available from the corresponding author, S.H.L., upon reasonable request.

## Supplementary Materials

The following supporting information can be downloaded at: https://www.mdpi.com/article/10.3390/ijms27031159/s1.
