# Supplementary material for: PrPC-Neutralizing Antibody Confers an Additive Benefit in Combination with 5-Fluorouracil in KRAS-Mutant Colorectal Cancer Models, Associated with Reduced RAS-GTP and AKT/ERK Phosphorylation
Source: Int J Mol Sci. 2026 Jan 23;27(3):1159. doi: 10.3390/ijms27031159 (PMC12897877; doi:10.3390/ijms27031159)
Supplement: Supplementary file 1 [file ijms-4081822-SI.pdf › ijms-4081822-SI.pdf]

(A)

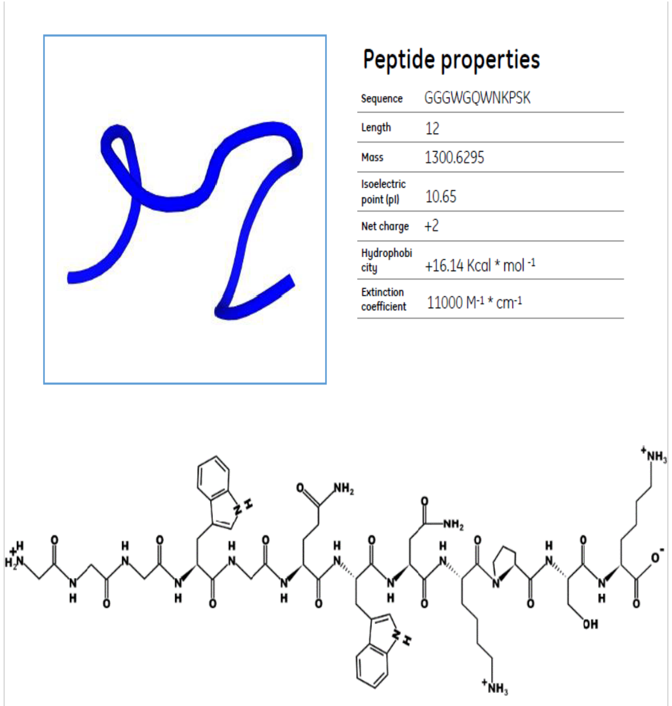

(B)

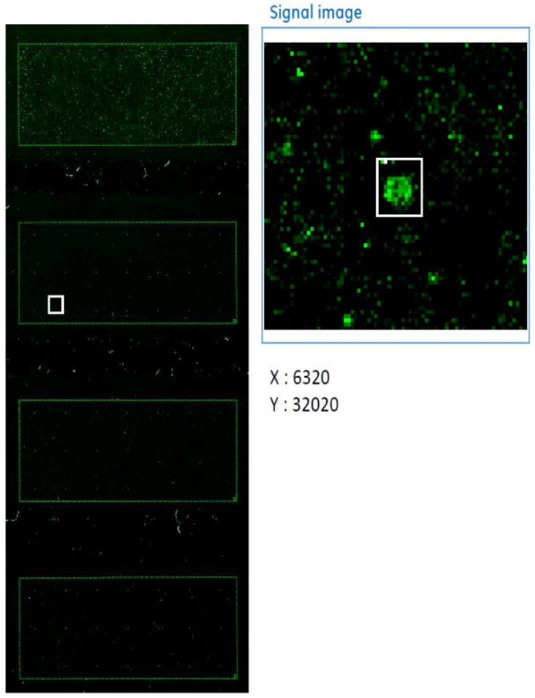

(C)

GGGWGQWNKPSK
GGSRYPWNKPSK
GGLGGYCKKRPK
GGNRYPWNKPSK
GGGGWGWNKPSK
MSRPIIWNKPSK
PKTNMKLISFLI
GWGQPHWNKPSK
KTNMKHTDVKMM
WGQPHGTNMKHM
CWMLVLWNKPSK
GGWNTGWNKPSK
LLISFLWNKPSK
FVATWSHSQWNK
WGQPHGWNKPSK
GNRYPPWNKPSK
ERESQAQRGSSM
GGSRYPCKKRPK
ILLISFKPSKPK
HGGGWGKPSKPK
YERESQGAAAAG
SQAYYQYRPMDE
CKKRPKPPQGGG
PVILLIWNKPSK
ISFLIFHSQWNK
HGGGWGNQVYYR
QPHGGGWNKPSK
FVATWSKPSKPK
PHGGGWHSQWNK

WNKPSK: 99-104

(D)

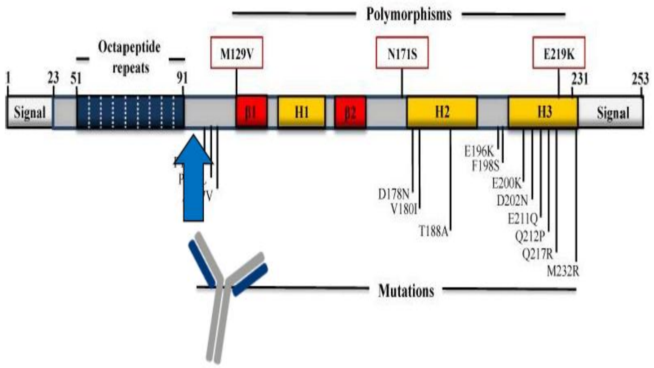

>sp|P04156|PRIO_HUMAN Major prion protein OS=Homo sapiens
MANLGCWMLVLFVATWSDLGLCKKRPKPGGWNTGGSRYPGQGSPGGNRYPPQGGGGWGQP 60
HGGGWGQPHGGGWGQPHGGGWGQPHGGGWGQGGGTHSQWNKPSKPKTNMKHMAGAAAAGA 120
VVGGLGGYMLGSAMSRPIIHFGSDYEDRYYRENMHRYPNQVYYRPMDEYSNQNNFVHDCV 180
NITIKQHTVTTTTKGENFTETDVKMMERVVEQMCITQYERESQAYYQRGSSMVLFSSPPV 240
ILLISFLIFLIVG

Supplementary Figure S1
